# Cross-Comparison of Leaching Strains Isolated from Two Different Regions: Chambishi and Dexing Copper Mines

**DOI:** 10.1155/2014/787034

**Published:** 2014-11-16

**Authors:** Baba Ngom, Yili Liang, Xueduan Liu

**Affiliations:** ^1^School of Minerals Processing and Bioengineering, Central South University, Changsha, Hunan 410083, China; ^2^Key Laboratory of Biohydrometallurgy, Ministry of Education, Changsha 410083, China

## Abstract

A cross-comparison of six strains isolated from two different regions, Chambishi copper mine (Zambia, Africa) and Dexing copper mine (China, Asia), was conducted to study the leaching efficiency of low grade copper ores. The strains belong to the three major species often encountered in bioleaching of copper sulfide ores under mesophilic conditions: *Acidithiobacillus ferrooxidans*, *Acidithiobacillus thiooxidans*, and *Leptospirillum ferriphilum*. Prior to their study in bioleaching, the different strains were characterized and compared at physiological level. The results revealed that, except for copper tolerance, strains within species presented almost similar physiological traits with slight advantages of Chambishi strains. However, in terms of leaching efficiency, native strains always achieved higher cell density and greater iron and copper extraction rates than the foreign microorganisms. In addition, microbial community analysis revealed that the different mixed cultures shared almost the same profile, and *At. ferrooxidans* strains always outcompeted the other strains.

## 1. Introduction

Bioleaching is the extraction of specific metals such as gold and copper from their ores through the exploitation of the oxidative activity of some microbial species, mainly sulfur and iron oxidizers [1]. Most of these ores are mineral sulfides categorized into “acid-soluble” and “acid-insoluble” metal sulfides. These two types of minerals are dissolved through two different pathways: thiosulfate pathway for acid-insoluble minerals and polysulfide pathway for acid-soluble metal sulfide ores [2, 3]. For both cases, leaching microbes provide the main leaching agents which consist of ferric iron and proton H^+^ [4, 5]. Over the last decades, a lot of work has been conducted on leaching microbes, and the descriptions of most of known microorganisms involved in biohydrometallurgy are well documented [4]. Several recent reviews have given a broad view of the microbial diversity within mining biotopes [6–8]. Even though huge efforts have been made on understanding such microorganisms, finding the suitable microorganisms or microbial combination, which may perform a high leaching efficiency and work under extreme environmental conditions, remains a huge challenge [9]. The efficiency is rather a result of the interaction of different microorganisms with different physiology than the action of a single microorganism [9]. However, physiological traits are not cumulative, and a microbial community composed of microorganisms with the most interesting physiological traits would not necessarily perform the best leaching efficiency in a given mineral ore [10]. Foreign strains such as commercially available microbial reference strains often prove ineffective for the leaching of new metal sulfides ores, due to their difficulty to adapt to and survive on the sulfides and other minerals present in an ore [11]. Therefore, more robust microbes and microbial combinations are being sought to enhance the bioleaching of these minerals. The use of native microbes, acclimatized to a given sulfide ore through many generations, may be one option for increasing viability and improving metal extraction efficiency. So this study aimed at investigating the leaching efficiency of native and foreign leaching microorganisms for the bioleaching of low grade copper ores.

For the purpose of this study, we compared some bacteria isolated from the Chambishi copper mine in Chambishi (Zambia) with those isolated from the Dexing copper mine (China). The strains we isolated belong to three major species commonly encountered in copper heap leaching operations:* Acidithiobacillus ferrooxidans* (an iron- and sulfur-oxidizing bacterium),* Leptospirillum ferriphilum* (a bacterium that only oxidizes iron), and* Acidithiobacillus thiooxidans* (a bacterium that only oxidizes reduced forms of sulfur). These three leaching bacteria have been reported to carry on various interactions (competition, synergism, and mutualism) in bioleaching systems and environment, and the strength of these interactions strongly impacts the global leaching efficiency [12].

This paper extends the study on microbial strains coming from different regions, to compare their physiological characteristics and their leaching efficiencies in their native and foreign ores. It also discusses whether the use of native microbial community is an alternative to overcome the ineffectiveness of available commercial reference strains in the bioleaching of new ores.

## 2. Materials and Methods

### 2.1. Bacterial Strains and Culture Conditions

Three strains including* Acidithiobacillus ferrooxidans* FOX1 (*Af* FOX1 accession number JF934687),* Leptospirillum ferriphilum* BN (*Lf* BN accession number JQ820324), and* Acidithiobacillus thiooxidans* ZMB (*At* ZMB accession number JQ820325) were isolated from Chambishi copper mine, Zambia.* Acidithiobacillus ferrooxidans* YTW (*Af* YTW accession number DQ062116),* Leptospirillum ferriphilum* YTW315 (*Lf* YTW315 accession number EU733647), and* Acidithiobacillus thiooxidans* (*At* A02 accession number FJ154540) were isolated from Dexing copper mine, China. Strains were isolated and/or purified by overlay plate (FeSo medium) [13]. The bacteria were cultured in 9K medium containing (NH_4_)_2_SO_4_ 3.0 g, KCl 0.1 g, K_2_HPO_4_ 0.5 g, MgSO_4_ *·*7H_2_O 0.5 g, and Ca(NO_3_)_2_ 0.01 g in 1 liter of water for 5 days. For* At. ferrooxidans* and* L. ferriphilum* strains, 9K was supplemented with 4.5% (wt/v) ferrous iron, while for* At. thiooxidans* 1% (wt/v) of sulfur was added. Growth and activity of the isolates under normal or various stress conditions, which include pH (1.2–3.0), temperature (25–45°C), heavy metals (CuSO_4_ (15–75 mmol L^−1^) and Fe_2_(SO_4_)_3_ (100–600 mmol L^−1^)), and organic compounds such as glucose, sucrose, yeast extract, peptone, and tryptone soya broth (0.1–1 g L^−1^), were examined in 9K medium supplemented with ferrous iron and sulfur.

### 2.2. Bioleaching Experiments

Low grade copper mineral samples were taken from Chambishi copper mine in Zambia (0.221% Cu) and Dexing copper mine in China (0.591% Cu). The copper moiety of Chambishi mineral sample was mostly composed of free copper oxide (72%), combined copper oxide (15%), and secondary copper sulfide (9.5%) while that of Dexing copper mine was mainly composed of primary copper sulfide (79%) (Table 1). For both mineral samples, the primary and copper sulfides were mainly composed of chalcopyrite and pyrite, respectively.

Bioleaching tests were carried out in 250 mL flasks containing 100 mL 9K medium without ferrous sulfate, under the following conditions: 30°C, initial pH 2.0, and 170 ×g. 9K basal salts medium supplemented with 3.0% (wt/v) pulp density was used. The inoculums were prepared by growing the microorganisms for 5 days in 500 mL conical flasks containing 200 mL of 9K medium. For bioleaching with mixed cultures, strains from Chambishi and those from Dexing were equally mixed (same number of cells) to form the Chambishi consortium (ZC) and the Dexing consortium (DC), respectively. In bioleaching, using Dexing ore sample, Dexing strains and Chambishi strains were considered as native and foreign bacteria, respectively, and vice versa for the Chambishi ore. Pure or mixed cultures were inoculated for a final cell density of approximately 10^6^ cells mL^−1^. Abiotic controls were also designed by replacing the bacterial inoculums by an equal volume of related medium. Aliquots of leachate were regularly taken, and redox potential, pH value, and iron and copper concentrations were analyzed. For the mixed cultures, bacterial compositions were analyzed based on quantitative PCR methods at the middle and the end of the experiment [14]. At the end of the bioleaching experiment, the leaching residues were washed, dried, and subjected to mineral analysis.

### 2.3. Analytical Methods

Chemical and mineral compositions of the mineral samples and leaching residues were analyzed by ICP-AES (IRIS Advantage 1000) and X-ray diffraction (XRD, D/MAX 2550 VB/PC, Rigaku, Japan), respectively. Ferrous and total iron concentrations in solution were determined by method of dichromate potassium [15] and UV-Vis molecular absorption spectrometry [16], while that of copper was determined by atomic absorption spectrophotometer (AAS). Iron and copper extraction rates were calculated as the ratio of total iron and total copper in leaching liquor to total iron and total copper initially present in the 3 g of mineral sample, respectively. The pH value was measured with pH S-3C acid meter, and the redox potential (or Eh) was determined with a platinum electrode with an Ag/AgCl reference electrode. Free cells in solution were observed and counted under an optical microscope. All the treatments were done in triplicate unless otherwise mentioned specifically, and the mean and standard deviation of the data from the three independent samples are presented.

## 3. Results and Discussion

### 3.1. Physiological Properties

Microorganisms used in bioleaching, like any other processes involving living beings, are persistently influenced by environmental, biological, and physicochemical factors, which affect bacterial activities and consequently the yield of metal extraction [17]. So in such work, it is of great importance to study the growth and activity of the isolates under normal or various stress conditions, including pH, temperature, and inhibitory substances such as low molecular weight organic matter and heavy metals.

**Figure 1:**
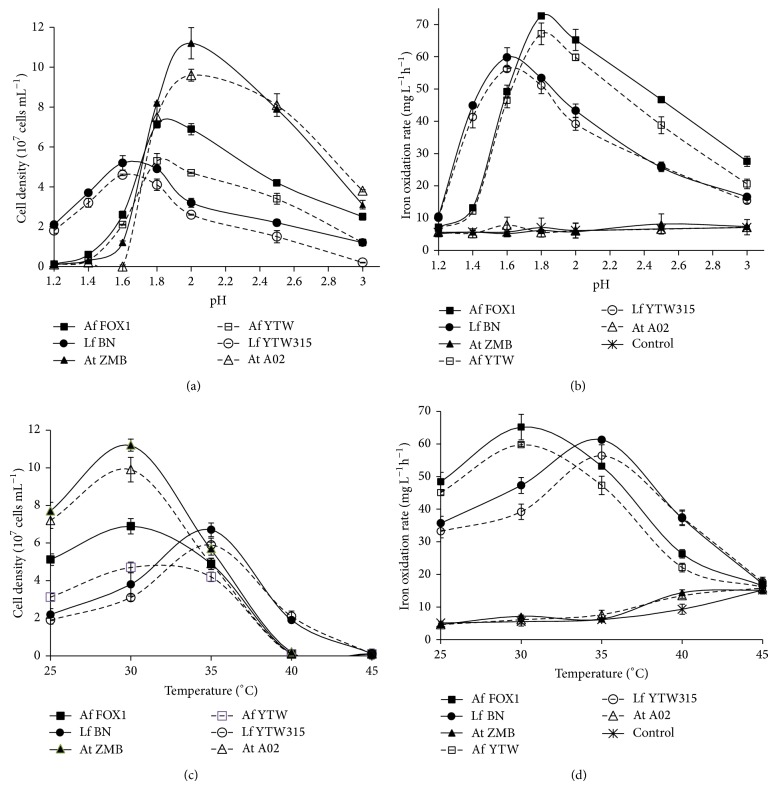
Influence of initial pH and temperature on cell growth and iron oxidation rates of the Chambishi strains (solid lines) and Dexing strains (long dash lines). Effect of pH on (a) cell growth and (b) iron oxidation rates of the isolates; effect of temperature on (c) cell growth and (d) iron oxidation rates of the different leaching strains.

In terms of energy source, the results revealed that* L. ferriphilum* strains were only iron-oxidizers and* At. thiooxidans* strains were only sulfur-oxidizers, while* At. ferrooxidans* could use both ferrous iron and sulfur. None of them could use organic substrates, but they were differently affected by the presence of carbohydrates, such as glucose, sucrose, yeast extract, peptone, and tryptone soya broth (see Table S1 in Supplementary Material available online at http://dx.doi.org/10.1155/2014/787034). For instance, strains of* At. ferrooxidans* were inhibited by glucose and sucrose but slightly stimulated by peptone and tryptone soya broth at concentration lower than 0.5 g L^−1^, while those of* L. ferriphilum* were inhibited by all these carbon substances. Similar results were reported by Patel et al. [18] who reported that* At. ferrooxidans* SRDSM2 responded to the addition of 0.5 g L^−1^ peptone and 1.0 g L^−1^ tryptone soya broth (TSB) in the ferrous sulfate tryptone soya broth (ITSB) medium with 35.3% and 29.6% increase in iron oxidation rate (IOR) but decrease in the IOR at higher peptone or tryptone soya broth levels [18]. This was more obvious with strain FOX1 (isolated from Chambishi) which scored an increase of IOR of 8.6% against 2.1% for strain YTW (isolated from Dexing) when 0.5 g L^−1^ TSB was added (see Figure S1 in Supplementary Material). From the obtained results, it is hard to speculate the mechanisms involved in this particular response of* At. ferrooxidans* strains on peptone and tryptone; however, it might be an interesting future research focus. Nevertheless this result confirms the higher tolerance of* At. ferrooxidans* species to organic matter and may explain the reason why members of* At. ferrooxidans* are much easier to grow on agar plate than* L. ferriphilum* strains [13, 19]. Again, for a given species, strains used the same energy source and responded similarly to the different organic compound; however, there was a slight difference in terms of metabolic activity. Strains isolated from Chambishi were found to have higher growth and iron oxidation rate. For instance,* At. ferrooxidans* FOX1 (isolated from Chambishi) and* At. ferrooxidans* YTW (isolated from China) showed an increase in cell density from ~10^6^ to 7.14 × 10^7^ and 5.23 × 10^7^ cells mL^−1^ (Figures 1(a) and 1(c)) with an iron oxidation rate of 72.6 and 67.1 mg L^-1 ^h^−1^ (Figures 1(b) and 1(d)) within 5 days in 9K medium containing 4.5% (w/v) of ferrous sulfate, respectively.

The influence of initial pH and temperature on the isolates' growth and metabolic activity was also determined. As can be seen from Figure 1,* L. ferriphilum* strains presented more interesting characteristics for their use in bioleaching, as they showed higher growth and iron oxidation rate at low pH and high temperature. At pH > 1.6,* At. ferrooxidans* scored a higher iron oxidation rate, but at pH < 1.6* L. ferriphilum* strains were found more efficient (Figure 1(b)). At temperature ranging between 30 and 35°C,* At. ferrooxidans* and* At. thiooxidans* presented higher cell density with higher activity, but around 40°C only growth of* L. ferriphilum* occurred (Figures 1(c) and 1(d)). This is important to be pointed out because these parameters impact not only the growth and activity of leaching bacteria but also the leaching kinetics of sulfide minerals [17]. By hindering ferric ion precipitation within the heap bed, low pH promotes heap permeability and the availability of the ferric ion reagent for the leaching of most sulfide minerals, whereas high temperature increases kinetics of chemical reaction involved in such mineral dissolution [20]. Again, under the same stress of pH and temperature, strains isolated from Chambishi showed higher activity, as noticed in normal condition (Figure 1).

**Figure 2:**
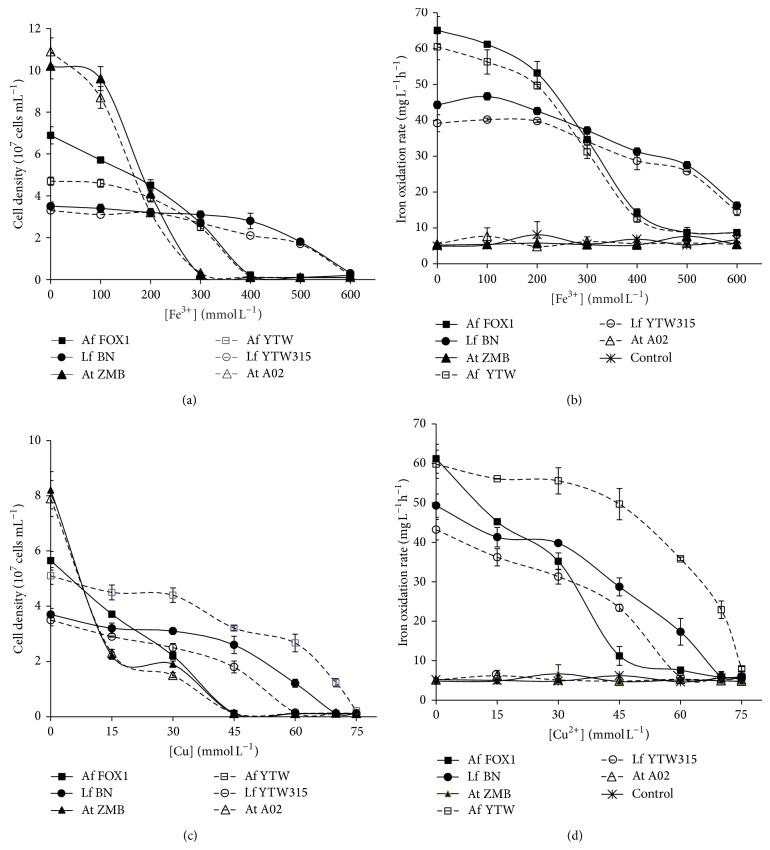
Influence of ferric iron and copper concentration on the growth and activity of the Chambishi strains (solid lines) and Dexing strains (long dash lines). Effect of ferric iron on (a) the growth and (b) the iron oxidation rates of the different strains; effect of copper on (c) the growth and (d) the iron oxidation rates of the strains.

Tolerance to metal ions (specifically ferric and copper ions) is an important factor for a leaching strain to be used in metal extraction and bioextraction of metals from copper mineral sulfide ores, as it results in dissolution of copper and iron as major soluble metals in the leachate; therefore, it was of interest to determine the influence of copper and ferric iron on the growth and activity of the strains. As can be seen from the results shown in Figure 2,* Leptospirillum ferriphilum* strains, whether they are from Chambishi or Dexing, both showed the highest tolerance to ferric ion. This result is consistent with many reports assuming that members of* Leptospirillum ferriphilum* species are less sensitive to ferric iron than those of* Acidithiobacillus ferrooxidans* and* Acidithiobacillus thiooxidans,* and at high redox potential (due to high ferric/ferrous iron ratio) they outcompete* Acidithiobacillus* genus [21, 22]. This property allows* Leptospirillum* sp. to play a pivotal role in mineral sulfide dissolution and specially during the late stage of metal sulfide leaching in which the high concentration of metal ions and Eh hinder the activity of leaching bacteria such as* At. ferrooxidans* species. In fact, at low temperature (30 to 40°C),* Acidithiobacillus ferrooxidans* and* Leptospirillum ferriphilum* are the main producers of ferric ions (1), which assures the chemical dissolution of metal sulfides (2), where Me represents metal ions:

(1) $$\begin{array}{c} 4{\text{Fe}}^{2+}+{\text{O}}_{2}+4{\text{H}}^{+}\overset{At.\;\;ferrooxidans}{\to }4{\text{Fe}}^{3+}+2{\text{H}}_{2}\text{O} \end{array}$$

(2) $$\begin{array}{c} \text{MeS}+{\text{Fe}}_{2}{\left(\text{S}{\text{O}}_{4}\right)}_{3}⟶\text{MeS}{\text{O}}_{4}+2{\text{FeSO}}_{4}+{\text{S}}^{\text{o}} \end{array}$$

So, not only do they have to perform a high iron oxidation rate, but they also have to be tolerant to ferric iron. In terms of comparison at origin level, there was not a big difference. For a given species, the strains presented almost the same tolerance to ferric iron, whether they are from Chambishi or Dexing.

Tolerance to copper is an important factor for the use of a strain in copper extraction from copper-bearing minerals, since the copper concentration of the pregnant solution can reach up to 100 mM [23]. Unlike ferric iron, tolerances to copper were slightly different among strains (Figure 2). For* At. ferrooxidans* strains, YTW (isolated from Dexing) presented a higher copper tolerance than FOX1 (isolated from Chambishi). In contrast, for* L. ferriphilum* isolates, that of Chambishi (strain BN) showed a higher copper tolerance than that of Dexing (YTW315). For* At. thiooxidans* strains, difference was not observed in terms of copper tolerance. Furthermore, unlike ferric ions, the copper resistance was not species-specific.

Basically, all the strains presented interesting properties that can be exploited in bioleaching of mineral sulfides; however, the conditions under which the physiological characterization was carried out were relatively simple and different from the complex conditions of leaching environment. So, a study of the strains in leaching environment would give a better insight into their leaching abilities.

### 3.2. Bioleaching Efficiency

**Figure 3:**
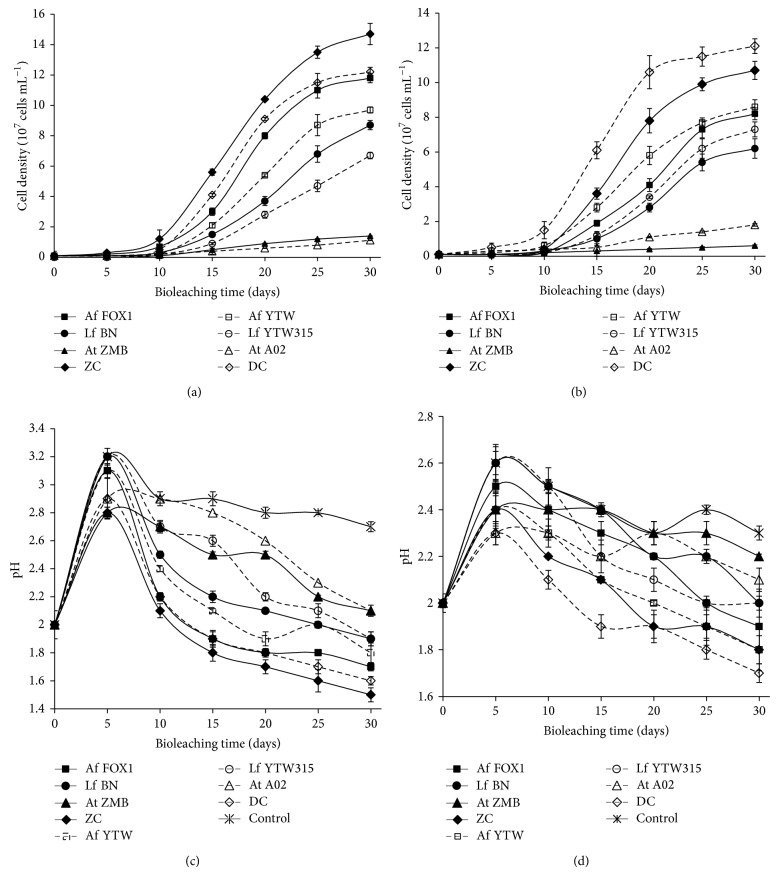
Changes in cell density and pH during bioleaching of low grade copper ores from Chambishi and Dexing with Chambishi strains (solid lines) and Dexing strains (long dash lines). Evolution of cell densities in pure and mixed cultures during the bioleaching of (a) Chambishi and (b) Dexing samples; changes in pH in pure and mixed cultures during the bioleaching of (c) Chambishi and (d) Dexing mineral samples.

The changes in pH and cell densities over time are shown in Figure 3. During the first 5 days, an increase in pH was observed in leaching liquor of both Dexing and Chambishi mineral samples; and sulfuric acid was added to maintain the pH at 2.0. This increase in pH is mainly due to the acid consumption by gangue minerals and the biological oxidation of Fe^2+^(1) [24]. After this period, a decrease in pH was observed, indicating the production of acids through the biological oxidation of elemental sulfur (3) or the precipitation of ferric ions ((4) and (5)).

Consider

(3) $$\begin{array}{c} 2{\text{S}}^{\text{o}}+3{\text{O}}_{2}+2{\text{H}}_{2}\text{O}\overset{At.\;\;thiooxidans}{\to }2{\text{H}}_{2}\text{S}{\text{O}}_{4} \end{array}$$

(4) 3Fe3++2SO42−+6H2O ⟶Fe3SO42OH6+6H+

(5) 3Fe3++2HSO4−+6H2O+M+ ⟶MFe3SO42OH6+8H+

where M^+^ = K^+^, Na^+^, or NH_4_^+^.

The decrease in pH was noticed in all samples, but it was much more important in systems with mixed culture followed by those with* At. ferrooxidans*. In fact,* At. thiooxidans* and, to some extent,* At. ferrooxidans* are the main producers of acid, through the biooxidation of sulfur (3), in mesophilic condition. So their copresence in mixed culture enhances the production of acid. For the systems with* At. thiooxidans*, less production of acid can be explained by the absence of sulfur produced through the chemical dissolution of metal sulfides by ferric iron, which cannot be regenerated by* At. thiooxidans*. In contrast, members of* Leptospirillum* genus can regenerate ferric iron but cannot oxidize sulfur compound released by mineral sulfide dissolution which explains the slight change in pH observed in systems inoculated with BN and YTW315. The decrease in pH in systems inoculated with* Leptospirillum* is rather a result of the precipitation of ferric iron ((4) and (5)) than that of sulfur biooxidation (3). In addition, the decrease in pH was much more important with native bacteria than foreign microorganisms, indicating higher activities of indigenous than foreign microbes. These results were also consistent with changes in Eh and cell densities. For instance, native microorganisms always achieved higher cell densities than foreign microorganisms (Figure 3). This result demonstrates the ease of native bacteria to adapt to their original ores [11].

**Figure 4:**
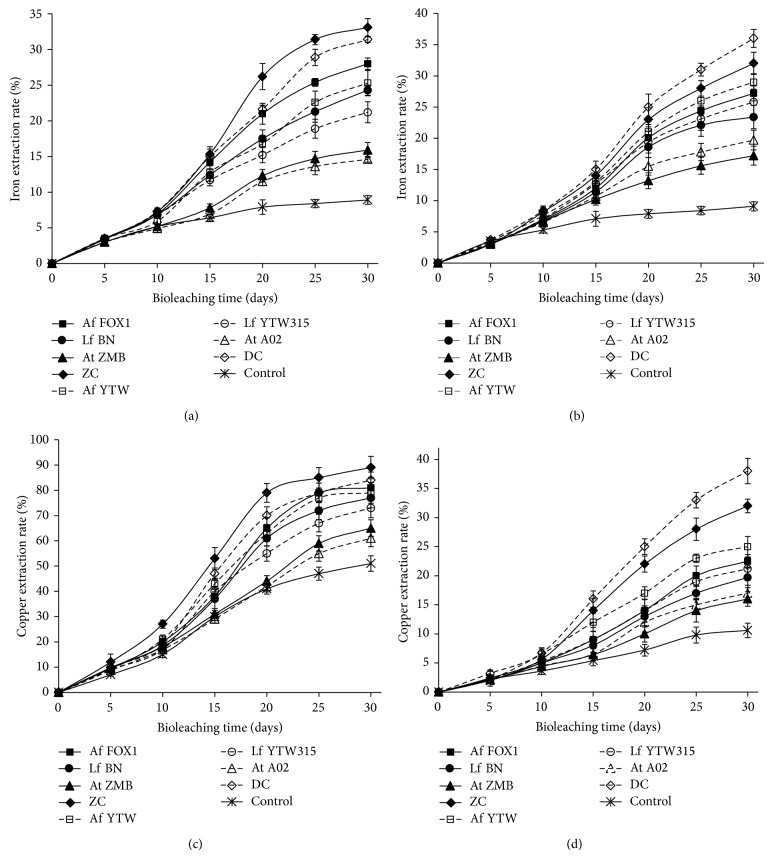
Iron and copper extraction rates during bioleaching of low grade copper ores from Chambishi and Dexing with Chambishi strains (solid lines) and Dexing strains (long dash lines). Iron extraction rates of (a) Chambishi and (b) Dexing mineral samples; copper extraction rates of (c) Chambishi and (d) Dexing mineral samples.

The iron and copper extraction rates of Dexing and Chambishi samples bioleaching are depicted in Figure 4. As can be seen, the iron and copper extraction rates followed the same trends, and they were consistent with changes in pH and cell densities (Figure 3), assuming that there are several interacting parameters in the bacterial leaching of sulfide minerals. Apart from being the relevant agent for metal sulfides dissolution, iron represents also an indicator for leaching efficiency, since it represents itself a product of most of the metal sulfide dissolutions (pyrite, chalcopyrite, and arsenopyrite). So, a high iron extraction rate leads to and indicates a high metal sulfide dissolution rate and consequently a high copper extraction rate. In other words, the iron and copper extraction rates are strongly correlated. Of course, in metal sulfide under the form of Cu_*x*_Fe_*y*_S_*z*_ such as chalcopyrite, this correlation is obvious; but even in metal sulfides in which copper and iron are not combined this correlation stands.

Figure 4 also shows that, for any samples, mixed cultures always performed the highest iron and copper extraction rates. In fact, it is the actions and interactions of different microorganisms with different abilities that provide high leaching efficiency rather than the action of a single microorganism (pure culture) [10]. Each of these three species detains some leaching abilities but not enough to give a high leaching efficiency. These results are in agreement with many reports suggesting that the iron oxidizers (*At. ferrooxidans* and* L. ferriphilum*) combined with sulfur oxidizers (*At. thiooxidans* and* At. caldus*) performed better than pure cultures of any of these three bacterial leaching species [25, 26].

Figure 4 reveals also that iron extraction rate was higher with native microorganisms than foreign strains. The same result was also observed in copper extraction rate, indicating that, for a given bacterial species or bacterial population composition, native microorganisms are always more efficient than foreign bacteria. Obviously, the microorganisms involved for setting in proper physicochemical changes like Eh, pH, temperature, and concentration of metals and metalloids into the system leading to mineral oxidation and dissolution are of particular significance [27]. However, the type of ores and the ease of the microorganism to adapt to it impact a lot the time that the leaching microorganisms take to create adequate mineral dissolution conditions [11, 27]. In the case of this study, foreign microorganisms were found to observe a longer lag phase than the native strains and consequently a delay in generating suitable conditions (high Eh and ferric iron concentration and low pH) for metal sulfide dissolution. These results demonstrate that the use of native microbes, even though the recovery of the suitable one is challenging, constitutes an option for increasing viability and improving metal extraction efficiency.

The composition of the copper moiety in the leaching residues of the Chambishi sample is presented in Table 2, while that of the Dexing mineral sample is shown in Table 3. Great changes have been noticed in the three forms of copper (free copper oxides, combined copper oxides, and secondary copper sulfides); however, the primary copper sulfide ratios in the residues were almost similar to those of the mineral samples (Tables 1, 2, and 3), suggesting that the latter was not effectively leached. It has been reported that, under mesophilic bioleaching conditions, primary copper sulfides, such as chalcopyrite, are often characterized by the slow dissolution kinetics and incomplete dissolution due to their refractory character, which is controversially attributed to the occurrence of a passivation layer on the mineral surface by elemental sulfur, ferric precipitates, or intermediate copper polysulfides [20, 25]. An effective bioleaching of such mineral sulfide requires high temperature [28] and low pH and consequently bacteria that can work under such condition (thermophilic or moderately thermophilic microorganisms). Considering the condition (30°C) under which the experiment was carried out, these results make sense; they are even in agreement with the copper extraction rate which revealed that the Chambishi ore sample (mostly composed of copper oxides and secondary copper sulfides) was easier to be leached than the Dexing ore sample (mainly composed of primary copper sulfides) (Figure 4).

### 3.3. Bacterial Population Analysis

**Figure 5:**
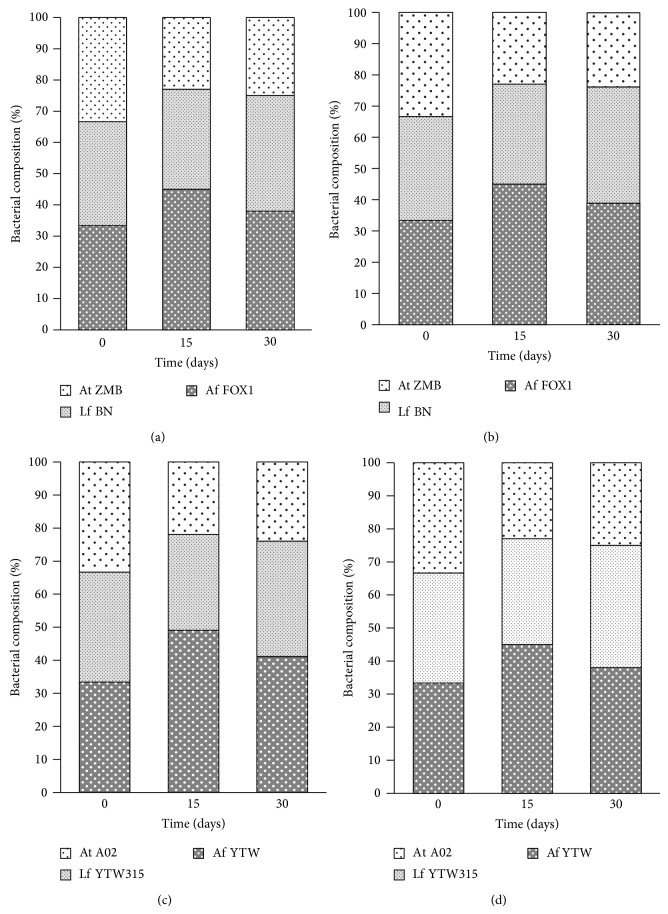
Changes in bacterial composition in mixed cultures systems. Bacterial composition of Chambishi consortium during the bioleaching of (a) Chambishi and (b) Dexing mineral samples; bacterial composition of Dexing consortium during the bioleaching of (c) Chambishi and (d) Dexing mineral samples.

Leaching bacteria do not only live in perfect symbiotic association in leaching environment; they may have a competition between them leading to changes in microbial population composition overtime. Changes in microbial population composition and structure are mainly according to the environmental conditions (pH, temperature, Eh, substrates, and inhibitors) which evolve also according to the microbial community activity. In this study,* At. ferrooxidans* strains (FOX1 and YTW) were found to be the dominant strains followed by* L. ferriphilum* strains (BN and YTW315) in all mixed cultures systems (Figure 5). For instance, in the bioleaching of the Chambishi sample with Chambishi strains, the percentage of* At. ferrooxidans* FOX1 increased from 33% to 47% during the first 15 days and then decreased to 40% on the 30th day. In contrast, that of* L. ferriphilum* BN decreased slightly during the first 15 days and then increased to ~37% on the 30th day.* At. thiooxidans* strains represented the lowest percentage. From 33%, their ratios decreased to 21% on the 15th day and remained almost the same (23%) at the end of the experiment. Similar results were also obtained with Dexing strains. These results are consistent with cells density in systems with pure culture, in which those inoculated with* At. ferrooxidans* strains always showed the highest cell density (Figure 3). Indeed, the condition under which the experiment was carried out is more suitable to* At. ferrooxidans* growth (compared to* L. ferriphilum*). Another reason is the versatility of members of this species in terms of substrate, unlike the two other species; members of* At. ferrooxidans* can use both ferrous ions and sulfur, and then have less susceptibility to encounter nutrient deficiency. In the last 10 days,* L. ferriphilum* strains tended to outcompete* At. ferrooxidans* isolates, and this was more obvious with bioleaching of Dexing mineral sample, in which the percentage of* L. ferriphilum* became almost equal to that of* At. ferrooxidans*. For instance, in system of the Dexing sample bioleaching with Dexing strains, the ratio of* At. ferrooxidans* and* L. ferriphilum* reached 38% and 37%, respectively (Figure 5). This phenomenon can be attributed to the increase in ferric iron [21, 22, 29]. Another explanation is the decrease of pH to which* At. ferrooxidans* is more sensitive than* L. ferriphilum* [7].

## 4. Conclusions

Physiological properties and leaching performances of six strains isolated from Chambishi (Chambishi) and Dexing (China) copper mines were characterized. The results revealed that, except for copper tolerance, strains within species presented almost similar physiological traits with slight advantages of Chambishi strains. Despite the slight physiological advantages of the Chambishi strains over the Dexing strains, the latter were more efficient for the bioleaching of the Dexing mineral sample, and vice versa. These results confirmed the fact that physiological characteristics are not cumulative, and a microbial community composed of microorganisms with the most interesting physiological traits would not necessarily provide the best leaching efficiency. This study also revealed that native strains were always more effective than foreign microorganisms for the bioleaching of a given ore, suggesting that the use of native microorganisms would be an interesting alternative to overcome the ineffectiveness of available commercial reference strains for the bioleaching of new mineral sulfide ores.

## Supplementary Material

Supplementary Material Table S1: This table is a selected biochemical and physiological characteristics of the new isolated strains. As expected, the new isolates are all mesophilic chemolitotroph; their optimal condition for growth range between 30 and 35°C, and they use ferrous ions (*At. ferrooxidans, L. ferriphilum* BN) and inorganic sulfur compounds (*At. thiooxidans*) as energy source and dioxide carbon as sole carbon source. None of them could use organic substrates, but they were differently affected by the presence of carbohydrates, such as glucose, sucrose, yeast extract, peptone, tryptone soya broth and organic acids (acetic and propionic acid). For instance, strains of *At. ferrooxidans* were inhibited by glucose and sucrose but stimulated by peptone and tryptone soya broth at concentration lower than 0.05% (wt/v), while that of *L. ferriphilum* were inhibited by all these carbon substances.
The isolates were also tested in high level ferric and copper ions concentrations. As expected, *L. ferriphilum* BN showed a higher tolerance to ferric ions (500 mM) than *At. ferrooxidans* FOX1 (300mM) and *At. thiooxidans* ZMB (200 mM). For copper tolerance also BN showed a higher copper tolerance than FOX1 (60 against 30 mM).
Figure S1: (it's Figure S1 and not Figure S2) This figure shows the response of iron oxidizers under different concentration of peptone and tryptone (0, 0.5 and 1.0 gL^−1^). *Leptospirillum ferriphilum* strains were inhibited with the increasing concentration of tryptone and peptone. In contrast, Acidithiobacillus ferrooxidans strains showed different responses according to the concentration of peptone and tryptone. The latter promoted the iron oxidation rate of *At. ferrooxidans* strains at concentration lower than 0.5 gL^−1^; while they inhibited the oxidation of ferrous ions at 1.0 gL^−1^.

## Figures and Tables

**Table 1:** Chemical and copper mineral composition (% wt/wt) of Chambishi and Dexing minerals samples.

Components	Chambishi	Dexing	Copper minerals	Chambishi	Dexing
Cu	0.221	0.591	Free copper oxide	**0.16**	0.03
Fe	1.991	6.612	Combined copper oxide	0.032	0.011
S	0.224	2.563	Secondary copper sulfide	0.021	0.08
As, Cd, Ni, Pb	<0.01	~0.01	Primary copper sulfide	0.008	**0.47**

			Total copper	0.221	**0.591**

**Table 2:** Copper mineral compositions (% wt/wt) of leaching residues of the Chambishi mineral sample after 30 days of bioleaching using X-ray diffraction (XRD).

Copper compounds	Systems								
	*Af* FOX1	*Lf* BN	*At* ZMB	ZC	*Af* YTW	*Lf* YTW315	*At* A02	DC	Control
Free copper oxide	0.01	0.014	0.034	0.003	0.012	0.016	0.035	0.007	0.035
Combined copper oxide	0.012	0.013	0.013	0.009	0.012	0.013	0.014	0.012	0.013
Secondary copper sulfide	0.011	0.012	0.018	0.006	0.013	0.015	0.017	0.011	0.022
Primary copper sulfide	0.007	0.007	0.008	0.006	0.007	0.007	0.009	0.005	0.009

Total copper	0.04	0.046	0.073	0.024	0.044	0.051	0.075	0.035	0.079

**Table 3:** Copper mineral compositions (% wt/wt) of leaching residues of the Dexing mineral sample after 30 days of bioleaching using X-ray diffraction (XRD).

Copper compounds	Systems								
	*Af* FOX1	*Lf* BN	*At* ZMB	ZC	*Af* YTW	*Lf* YTW315	*At* A02	DC	Control
Free copper oxide	—	—	—	—	—	—	—	—	—
Combined copper oxide	—	—	—	—	—	—	—	—	—
Secondary copper sulfide	0.041	0.037	0.047	0.023	0.035	0.038	0.041	0.018	0.057
Primary copper sulfide	0.416	0.436	0.453	0.367	0.413	0.427	0.449	0.354	0.463

Total copper	0.457	0.473	0.496	0.389	0.448	0.465	0.490	0.372	0.523
