# Supplementary material for: Cross-Comparison of Leaching Strains Isolated from Two Different Regions: Chambishi and Dexing Copper Mines
Source: Biomed Res Int. 2014 Nov 16;2014:787034. doi: 10.1155/2014/787034 (PMC4248400; doi:10.1155/2014/787034)
Supplement: Supplementary file 1 — Supplementary Material Table S1: This table is a selected biochemical and physiological characteristics of the new isolated strains. As expected, the new isolates are all mesophilic chemolitotroph; their optimal condition for growth range between 30 and 35°C, and they use ferrous ions (At. ferrooxidans, L. ferriphilum BN) and inorganic sulfur compounds (At. thiooxidans) as energy source and dioxide carbon as sole carbon source. None of them could use organic substrates, but they were differently affected by the presence of carbohydrates, such as glucose, sucrose, yeast extract, peptone, tryptone soya broth and organic acids (acetic and propionic acid). For instance, strains of At. ferrooxidans were inhibited by glucose and sucrose but stimulated by peptone and tryptone soya broth at concentration lower than 0.05% (wt/v), while that of L. ferriphilum were inhibited by all these carbon substances. The isolates were also tested in high level ferric and copper ions concentrations. As expected, L. ferriphilum BN showed a higher tolerance to ferric ions (500 mM) than At. ferrooxidans FOX1 (300mM) and At. thiooxidans ZMB (200 mM). For copper tolerance also BN showed a higher copper tolerance than FOX1 (60 against 30 mM). Figure S1: (it's Figure S1 and not Figure S2) This figure shows the response of iron oxidizers under different concentration of peptone and tryptone (0, 0.5 and 1.0 gL−1). Leptospirillum ferriphilum strains were inhibited with the increasing concentration of tryptone and peptone. In contrast, Acidithiobacillus ferrooxidans strains showed different responses according to the concentration of peptone and tryptone. The latter promoted the iron oxidation rate of At. ferrooxidans strains at concentration lower than 0.5 gL−1; while they inhibited the oxidation of ferrous ions at 1.0 gL−1. [file 787034.f1.docx]

**Table S1 Selected biochemical and physiological characteristics of the new isolates**

| Isolates  Characteristics | *At. ferrooxidans* FOX1 | *At. thiooxidans* ZMB | *L. ferriphilum* BN |
| --- | --- | --- | --- |
| Optimal Temp (^o^C) | 30 | 30 | 35 |
| Optimal pH | 1.8 | 2 | 1.6 |
| Growth | A | A | A |
| Growth on Ferrous iron | ++ | - | ++ |
| Growth on Sulfur | + | ++ | - |
| Growth on MS | + | + | + |
| Growth on Glucose | -- | -- | -- |
| Growth on Sucrose | -- | -- | -- |
| Growth on Tryptone | +/- | -- | -- |
| Growth on Peptone | +/- | -- | -- |
| Growth on Organic acid | -- | -- | -- |
| Copper tolerance (mM) | 30 | 30 | 60 |
| Ferric tolerance (mM) | 300 | 200 | 500 |

MS = Metal sulfides; A = autotroph; H = heterotroph; mM = mmol/L; (++) = higher growth rate; (+) = positive; (-) = negative; (--) = Inhibition effect; (+/-) = stimulation effect at low concentration (<0.05% wt/v) and inhibition effect at relatively high concentration (>0.1% wt/v).


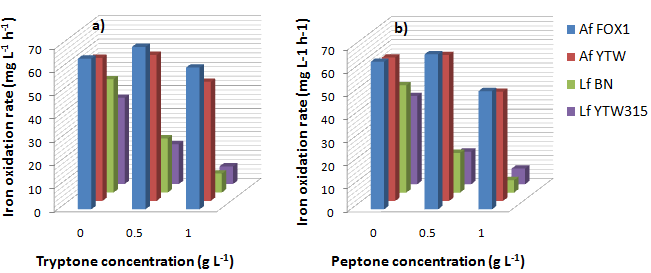


Fig. S1 Influence of (a) tryptone and (b) peptone on iron oxidation rate of Acidithiobacillus ferrooxidans and Leptospirillum ferriphilum strains
